# Robotic natural-orifice IntraCorporeal anastomosis with Extraction (NICE procedure) for complicated diverticulitis

**DOI:** 10.1007/s00464-021-08350-z

**Published:** 2021-02-22

**Authors:** Eric M. Haas, Thais Reif de Paula, Roberto Luna-Saracho, Melissa Sara Smith, Jean-Paul J. LeFave

**Affiliations:** 1 Houston Colon PLLC, Houston, TX USA; 2 Division of Colon and Rectal Surgery, Houston Methodist Hospital, 6560 Fannin Street, Suite #1404, Houston, TX 77030 USA; 3 Department of Biomedical Sciences, University of Houston, Houston, TX USA

**Keywords:** Intracorporeal anastomosis, Natural-orifice specimen extraction, NICE procedure, Diverticulitis, Colorectal surgery, Minimally invasive surgery

## Abstract

**Background:**

Totally intracorporeal surgery for left-sided resection carries numerous potential advantages by avoiding crossing staple lines and eliminating the need for an abdominal incision. For those with complicated diverticulitis, minimally invasive surgery is known to be technically challenging due to inflamed tissue, distorted pelvic anatomy, and obliterated tissue planes, resulting in high conversion rates. We aim to illustrate the stepwise approach and modifications required to successful complete the robotic Natural-orifice IntraCorporeal anastomosis with transrectal specimen Extraction (NICE) procedure in this cohort.

**Methods:**

Consecutive, elective, unselected patients presenting with complicated diverticulitis defined as fistula, abscess and stricture underwent the NICE procedure over a 24-month period. Demographic and intraoperative data were collected, and video recordings were reviewed and edited on encrypted server.

**Results:**

A total of 60 patients (50% female) underwent the NICE procedure for complicated diverticulitis with a mean age of 58.9 years and mean BMI of 30.7 kg/m^2^. The mean operative time was 231.6 min. All cases (100%) were achieved with intracorporeal anastomosis using a circular stapling device. All but one patient (98.3%) had successful transrectal extraction of the specimen. Forty-four (73%) of the specimens required a specimen-thinning maneuver to successfully extract the specimen and there were no conversions. We identified seven key technical modifications and considerations to facilitate successful completion of the procedure which are illustrated, including early release of the disease, mesentery-sparing dissection, dual instrument control of the mesenteric vasculature, release of the rectal reflection, use of NICE back table, specimen-thinning maneuver, and closure of the rectal cuff.

**Conclusion:**

We present a stepwise approach with key modifications to successfully achieve totally robotic intracorporeal resection for those presenting with complicated diverticulitis. This approach may help overcome the technical challenges and provide a foundation for reproducible results.

**Supplementary Information:**

The online version contains supplementary material available at 10.1007/s00464-021-08350-z.

First described in laparoscopic surgery over 25 years ago [1, 2], colorectal resection with intracorporeal anastomosis (ICA) and transrectal extraction of specimen (TRSE) has not gained significant traction. Early experience revealed numerous patient benefits including less postoperative pain, less opioid utilization, faster recovery, lower complication rates and greatly reduced hernia rates compared to conventional laparoscopy [3–5]. Yet it is estimated that this approach is offered to fewer than 1% patients presenting for a left-sided colorectal resection, primarily due to technical barriers [6].

Enabling technologies such as the robotic platform, as well as a continued push toward total intracorporeal surgery has resulted in a resurgence of intertest. Robotic right sided procedures with ICA for instance have been gaining widespread adaptation [7] but eliminating the extraction incision altogether for procedures involving the left colon has yet to be examined in earnest. We first reported on the feasibility of robotic natural-orifice ICA with transrectal extraction of the specimen for left colectomy in 2018 and termed it the NICE procedure [6]. Thereafter we reported a stepwise technique to afford a reproducible and consistent approach [8].

We have since expanded our utilization of the robotic NICE procedure to more technically challenging cases involving complicated diverticulitis. These procedures are known to involve an inflammatory process with thickened mesentery, distorted pelvic anatomy, obliterated tissue planes and high conversion rates [9–11]. As such, modifications and refinements of the technique are necessary to successfully complete the NICE approach.

This study and video describe and illustrate key steps, surgical maneuvers and modifications that we have adopted over the course of consecutive cases to safely and successfully complete the NICE procedure for complicated diverticulitis. This stepwise approach affords reproducibility and consistency to help overcome the technical challenges.

## Methods

Case videos were recorded on a secure and encrypted server and under IRB protocol. All procedures were performed utilizing the da Vinci Xi platform (Intuitive Surgical, Sunnyvale, CA, USA). From June 2018 to May 2020, a total of 134 consecutive elective surgical resections with primary anastomosis for diverticulitis was performed. This study consists of the subset of 60 of these patients who presented with complicated diverticulitis defined as the presence of a fistula, stricture and/or abscess at the time of surgery. The presence of abscess was confirmed in all cases with positive intraoperative cultures. Preoperative workup included confirmatory imaging as well as colonoscopy within the appropriate timeframe to confirm the presence of diverticular disease and ensure the absence of colorectal cancer or other pathology. The procedures were performed by an experienced board-certified colorectal surgeon (EMH) in one of two institutions in Texas Medical Center (Houston Methodist Hospital and Baylor St Luke’s Medical Center in Houston, Texas).

### Study variables and outcome measures

Demographic data included age, gender, BMI, and American Society of Anesthesiologists (ASA) classification. Disease and operative data included diagnosis, surgical procedure, operative time, estimated blood loss (EBL), number and size of ports, splenic flexure takedown, type of anastomosis, completion of ICA, completion of transrectal extraction of specimen, and intraoperative complications. Data were collected from the electronic medical records, recorded and entered into a study protocol approved by the internal review board (IRB) (study protocol 00012111). Data were reported as frequencies (percentages) for categorical data and mean (standard deviation) and range, for continuous data. A Chi-square test, or Fisher’s exact test, as appropriate, were used for analysis of categorical data and independent *t*-test was used for continuous data. All comparisons were two sided, and statistical significance was defined as *p* < 0.05. All analysis was performed with SPSS version 26 (IBM Corp. Armonk, NY, USA).

### Video recordings

The procedures were recorded and analyzed. The defined steps of the NICE procedure have been previously described [6, 8]. Surgical maneuvers and modifications specifically addressing complicated diverticulitis are additionally described below.

**Fig. 1:**
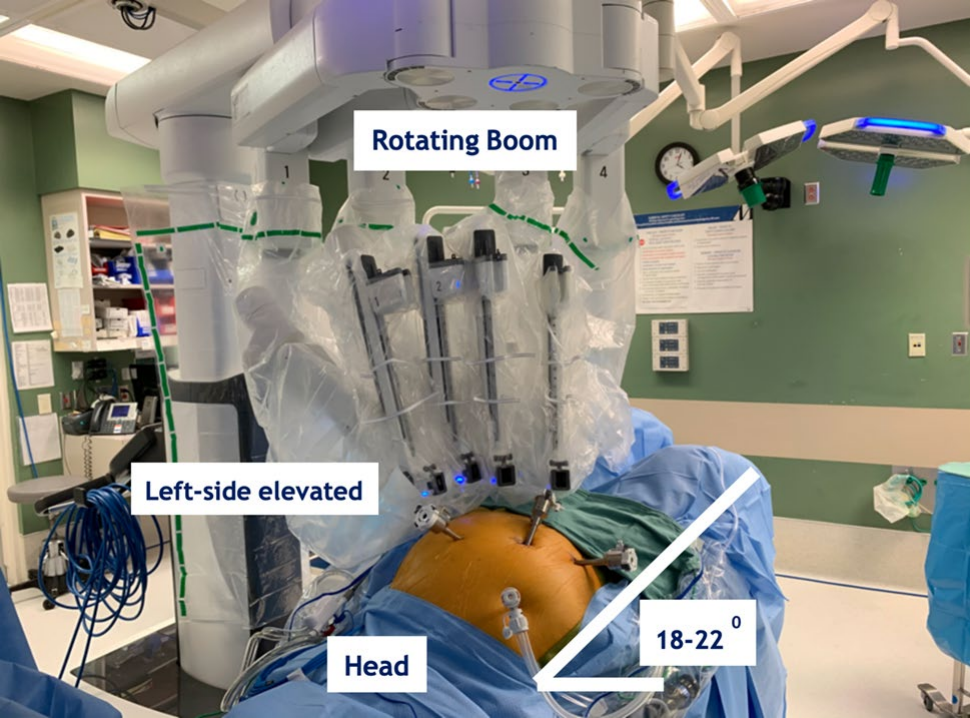
Patient positioning

**Fig. 2:**
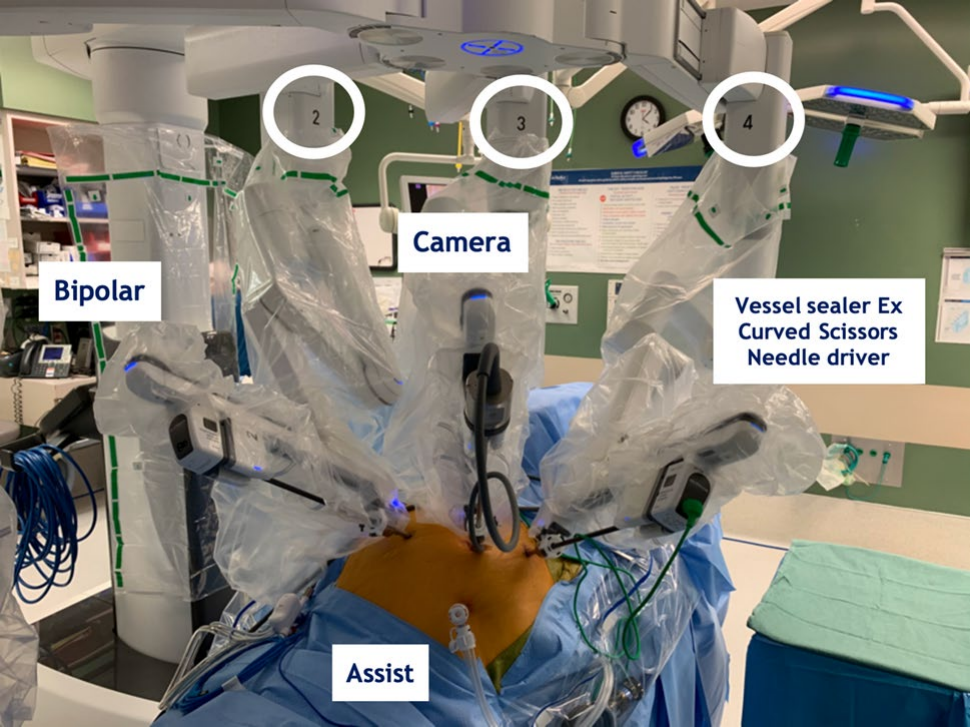
Ports placement and docking

Briefly, the NICE procedure was performed with the patient placed in 18–22 degrees of Trendelenburg with 8 degrees of right tilt (left side elevation) (Fig. 1). We begin the procedure with optical entry (Optiview, Inc., Jacksonville, FL, USA) in the right upper quadrant (RUQ) and exchange with a 5 mm AirSeal® (ConMed Corporation, Utica, NY, USA). This RUQ access facilitates tissue retraction, suction and passing sutures by the bedside assistant. We then place the following ports: 8 mm robotic port in the right lower quadrant (RLQ), 8 mm robotic port in the umbilicus, and an 8 mm robotic port in the left upper quadrant (LUQ). The robotic boom is positioned over the left hip and robotic arm number 2 is docked to the LUQ port for use of a fenestrated bipolar, robotic arm 3 at the umbilicus for the camera, and robotic arm 4 in the RLQ for the Vessel Sealer Extend (VSE) (Intuitive Surgical Inc., Sunnyvale, CA, USA) and exchanged for the curved monopolar scissors or needle driver throughout the procedure (Fig. 2). In some cases, a 4th robotic arm is required for deep pelvic exposure and is placed in the left lower or upper quadrant for the Cadiere forceps.

We draw attention to the sigmoid colon and begin dissection in a lateral to medial fashion along the white line of Toldt in a cranial direction along the left colon. As a standard, we proceed with splenic flexure takedown by dividing the splenocolic ligament and entering the lesser sac. We then turn our attention to the area of disease in the pelvis and release the bowel from the peritoneal adhesions along the pelvic side wall. When present we then enter into the abscess cavity and drain it and/or release the fistula from its point of origin to the bladder, uterus or vagina by dropping down the disease. In preparation for the transrectal extraction, we release the left and right lateral rectal reflection as well as the anterior refection.

We turn our attention to the proposed proximal resection margin at the level of the left and sigmoid colon and develop a window in the mesentery. Mesenteric-sparing division is achieved without entering the retroperitoneum and preserving the superior rectal artery. Using the VSE in combination with the fenestrated bipolar, we divide the mesentery close to the bowel in a cranial to caudal direction until the upper third of the rectum is reached. The bowel is divided at the proximal and distal levels of resection using the VSE along with the suction nearby to aspirate any leakage of stool contents.

**Fig. 3:**
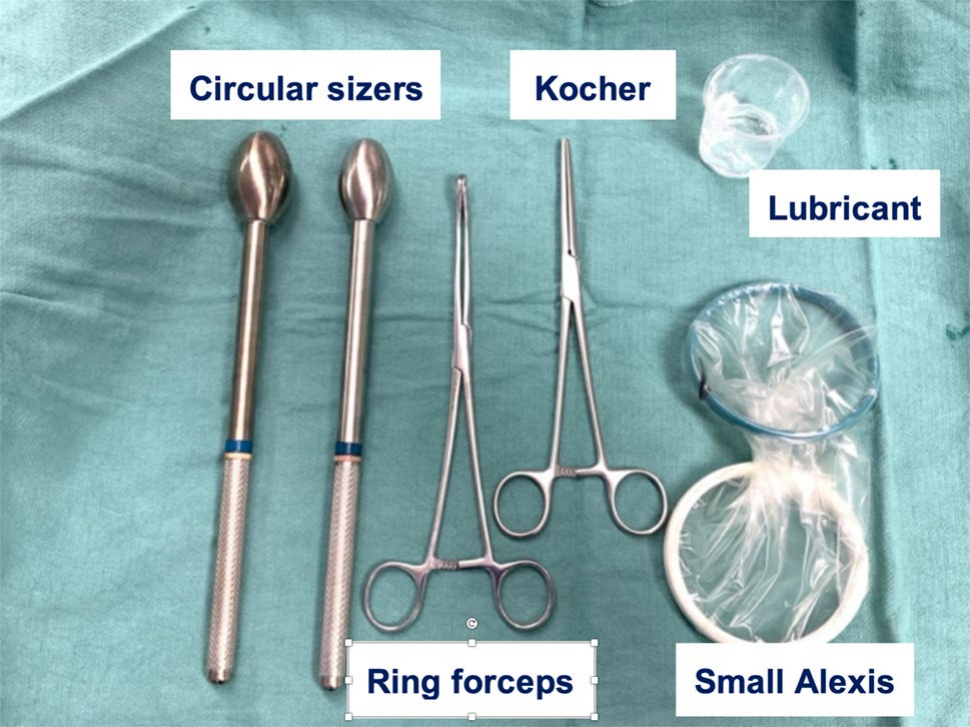
NICE back table

**Fig. 4:**
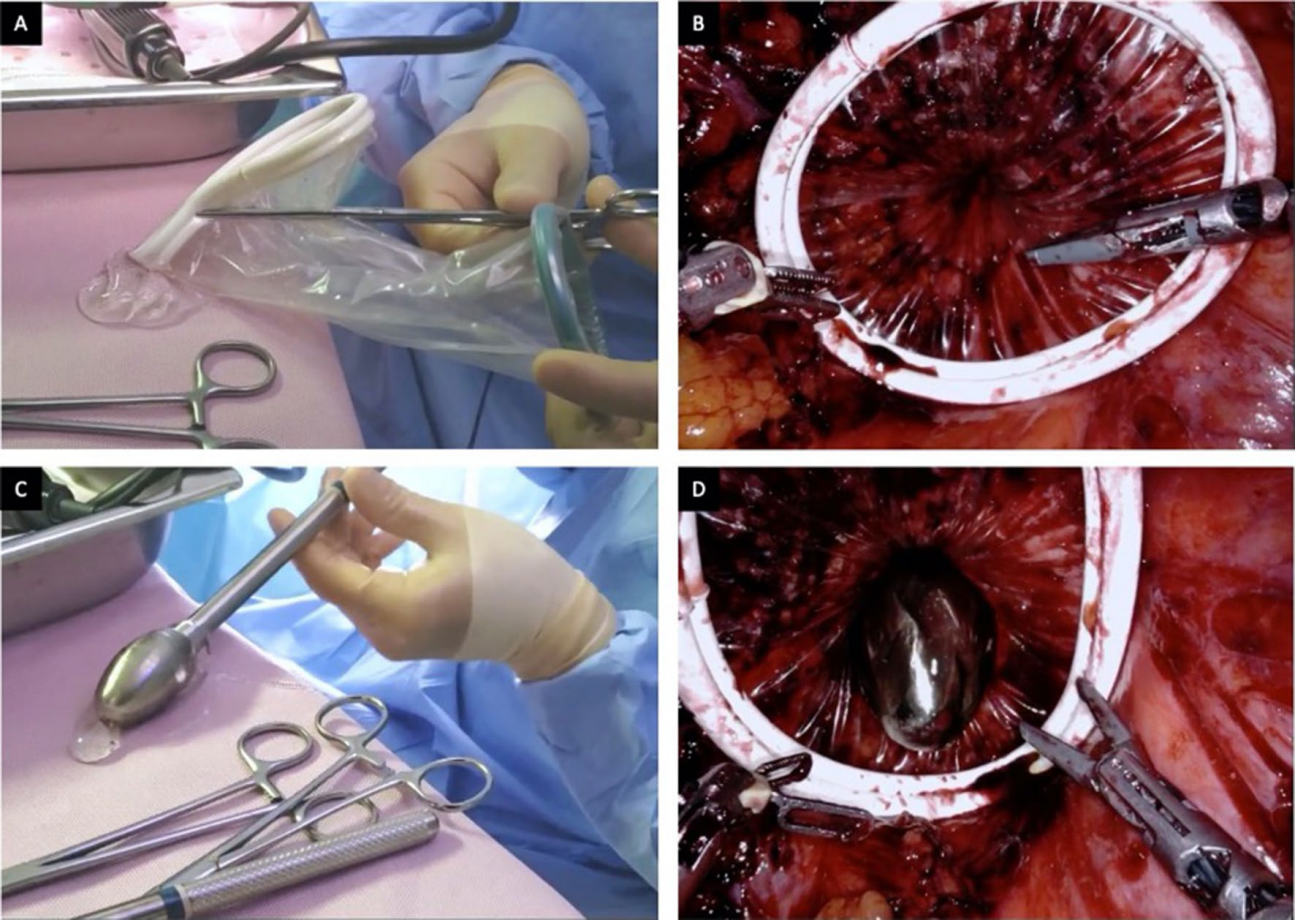
**A** Alexis preparation and lubrication. **B** Alexis inserted transrectally, in place for extraction phase. **C** Circular sizer preparation and lubrication. **D** Dilation of sphincters with circular sizer previously to extraction of specimen(s)

In preparation of the natural-orifice steps of the procedure, as assistant surgeon uses a NICE back table as shown in Fig. 3. A small sized Alexis wound retractor (Applied Medical, Rancho Santa Margarita, CA, USA) is clamped with a Kocher, lubricated and carefully inserted transanally and expands across the divided rectal wall. The rectal lumen is then dilated with a medium and large circular sizer (Fig. 4).

**Fig. 5:**
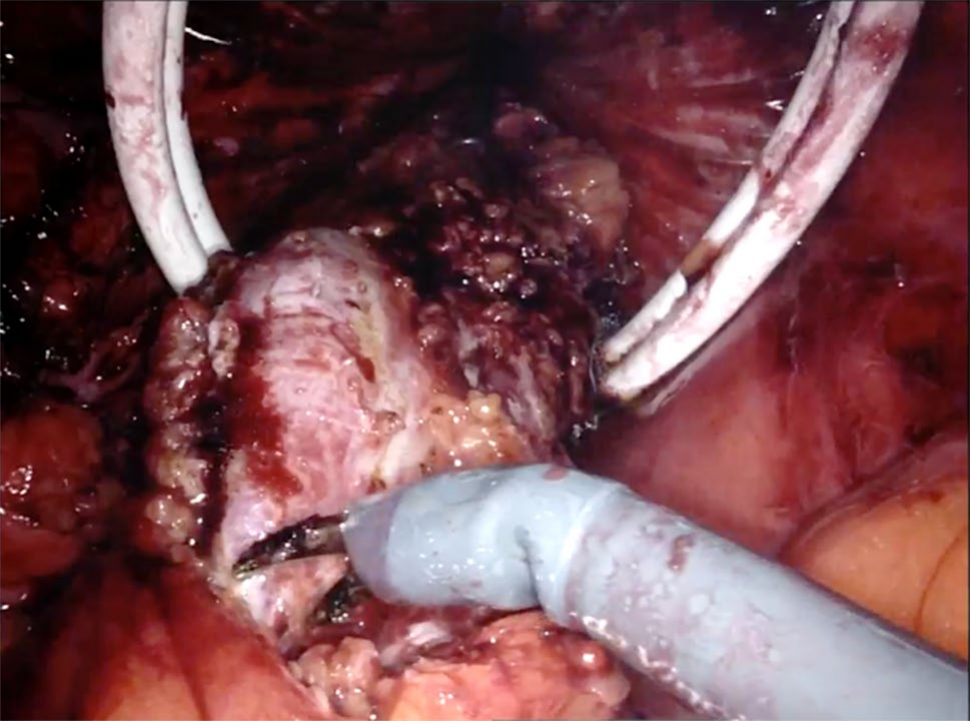
Shaving of the bowel wall mesentery

**Fig. 6:**
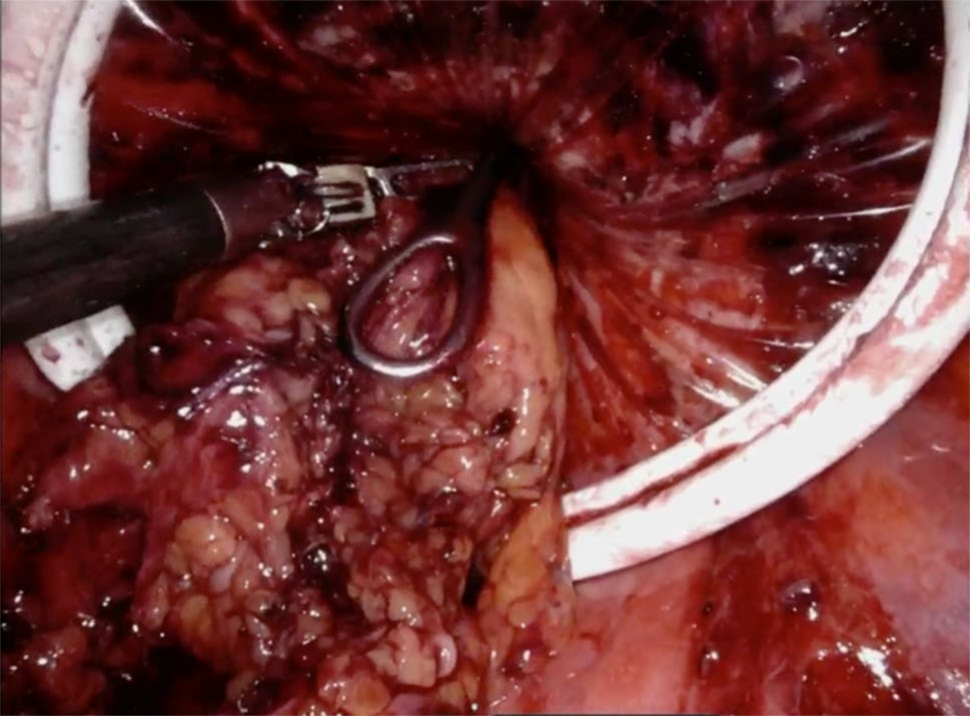
Extraction of the mesentery

**Fig. 7:**
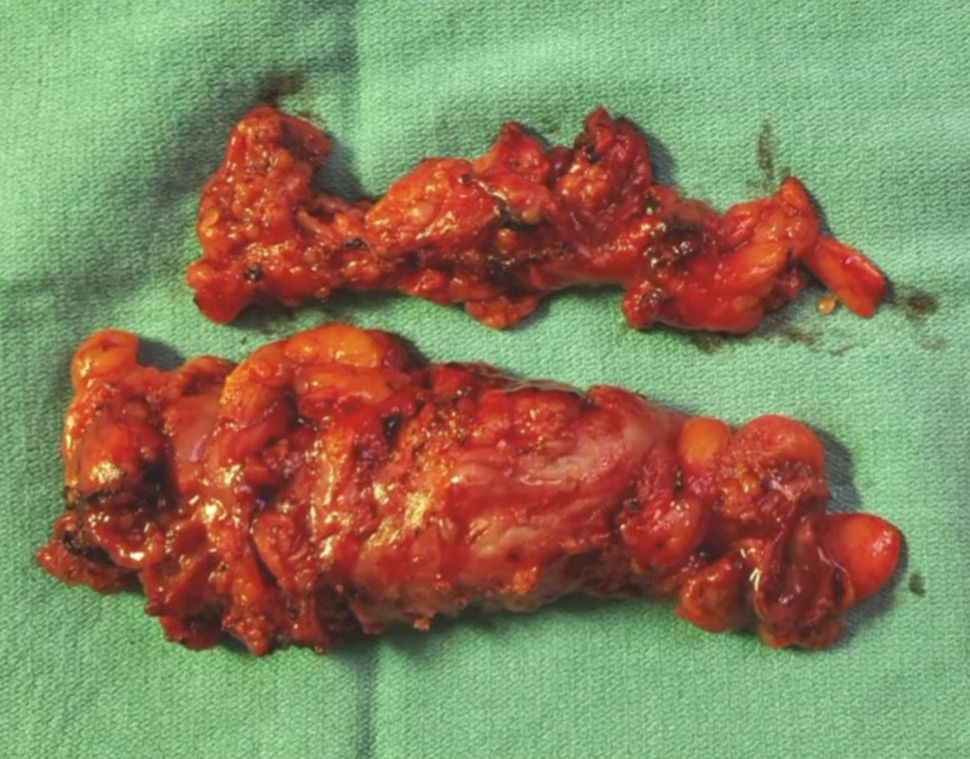
Rectosigmoid segment separated from the mesentery

A long ring forceps is then inserted through the Alexis and grasps the divided edge of the specimen for extraction. For large specimens with a bulky mesentery, trauma during the extraction process is mitigated by thinning the specimen prior to extraction. The monopolar scissors are used to release the mesentery along the length of the bowel in a linear fashion while under traction (Fig. 5). The specimen is then extracted transrectally (Figs. 6, 7).

In preparation for the ICA, the Alexis is inverted and removed, and the circular stapler device is introduced transrectally. The anvil is detached and then it is secured to left colon with a pursestring suture consisting of 6-inch, 3.0 barbed suture on a V20 needle (V-Loc 180™, Covidien; Mansfield, MA, USA). In the event that bowel contents have leaked from the open colon during the extraction process, the area is locally irrigated and aspirated until clear. The rectal cuff is closed around the spike of the stapler with a second pursestring suture to facilitate the colorectal anastomosis. We routinely use Firefly® (Intuitive Surgical Inc., Sunnyvale, CA, USA) perfusion assessment prior to performing the anastomosis. In cases in which the distal transection is in the lower rectum, the robotic stapler is used to close the rectal cuff.

### Postoperative management

All patients received enhanced recovery pathways (ERAS) postoperatively, consisting of early ambulation, education and counseling, early feeding and multimodal opioid-sparing pain control recovery.

## Results

Of a total of 134 consecutive cases of diverticulitis, 60 (44.8%) presented with complicated disease and were evaluated in this study. Half (50%) of the cohort were female and nearly half (46.7%) of the patients were ASA III. The mean age was 58.9 years old (range 22–84) and the mean BMI was 30.7 kg/m^2^ (range 19–37.6). Half (50%) of the cohort had a BMI ≥ 30 kg/m^2^ (*n* = 30), and 25% had a BMI ≥ 35 kg/m^2^ (*n* = 15). Thirty-two (53.3%) patients had a history of prior abdominal surgery. Complicated diverticulitis was defined as the presence of a fistula with or without abscess in 35 patients (58%), abscess cavity alone in 18 cases (30%), and stricture with or without fistula or abscess in 7 patients (12%). A comparison of the demographic features among patients presenting with complicated and uncomplicated disease is summarized in Table 1.

**Table 1:** Demographics and patient characteristics

	Complicated diverticulitis (*n* = 60)	Uncomplicated diverticulitis (*n* = 74)	*p*-value
Age in years, mean ± SD (range)	58.9 ± 13.6 (22–84)	58 ± 11.7 (34–81)	0.684^b^
Gender, no. (%)			
Female	30 (50.0%)	40 (54.1%)	0.640^a^
Male	30 (50.0%)	34 (45.9%)	
BMI (kg/m^2^)			
Mean BMI ± SD (range)	30.7 ± 6.2 (19–47.6)	28.3 ± 4.2 (20–40)	0.011^b^
BMI ≥ 30, no. (%)	30 (50)	24 (32.4)	
ASA, no. (%)			
I–II	32 (53.3)	48 (64.9%)	0.142^a^
III	28 (46.7)	26 (35.1%)	
Previous abdominal surgery, no. (%)			
Yes	32 (53.3)	32 (45.7)	0.834^a^
No	28 (46.7)	38 (54.3)	
Diagnosis, no. (%)			
Fistula	35 (58)	0 (0)	< 0.001^c^
Abscess	18 (30)	0 (0)	
Stricture	7 (12)	0 (0)	

*SD* standard deviation, *BMI* body mass index, ASA American Society of Anesthesiology

^a^Pearson’s Chi-square

^b^Independent *t*-test

^c^Fisher’s exact test

The mean operative time was longer in the complicated diverticulitis cohort, 231.6 ± 75.0 vs 194.9 ± 53.7 min (*p* = 0.004). In the complicated diverticulitis cohort, the mean estimated blood loss was 59.2 ml (range 10–250) and one patient required intraoperative blood transfusion. A 5 mm accessory assistant port was utilized in all cases. In 65.5% of the cases, we utilized three 8 mm ports and in 34.5% we utilized a fourth robotic 8 mm port. We replaced the right lower quadrant 8 mm port with a 12 mm port in 8 cases (13.3%) for use of the robotic stapler.

In the complicated diverticulitis cohort, all cases (100%) were achieved with ICA using a circular stapling device. The anvil was secured with a pursestring suture and an endoloop to the proximal colon in all cases (100%). In 86.7% of the cases, the rectal cuff was closed with pursestring suture and an anastomosis was formed without any crossing staple lines. In 13.3% of cases, the distal resection was in the low rectum and the rectal cuff was closed with the robotic stapler in preparation for the anastomosis. All but one patient (98.3%) had successful transrectal extraction of the specimen. This patient presented with near-obstructing disease in which we could not definitively rule out malignancy prior to surgery and therefore did not opt for transrectal extraction. Forty-four (73%) of the specimens required a specimen-thinning maneuver, consisting of shaving the mesentery from the colon wall to successfully extract the specimen. Splenic flexure takedown was performed in all but one patient (98.3%). A diverting loop ileostomy was created in 11 (18%) patients, all of whom had perforating disease with the presence of an abscess. There were three cases in which we identified a partial-thickness tear along the anterior wall of the rectum after the transrectal extraction of the specimen. In each case, we repaired it without complication or diversion. There were no conversions. The remaining intraoperative outcomes were similar across cohort and are outlined in Table 2.

**Table 2:** Intraoperative outcomes

	Complicated diverticulitis (*n* = 60)	Uncomplicated diverticulitis (*n* = 74)	
Operative time in minutes, mean ± SD (range)	231.6 ± 75.0 (126–443)	194.9 ± 53.7 (107–449)	0.004^b^
Estimated blood loss in ml, mean ± SD (range)	59.2 ± 51.0 (10–250)	48.7 ± 42.8 (5–300)	0.198^b^
Intraoperative transfusion, no. (%)			
Yes	1 (1.7)	0 (0)	0.438^c^
No	59 (98.3)	74 (100)	
Splenic flexure takedown performed, no. (%)			
Yes	59 (98.3)	68 (91.9)	0.096^a^
No	1 (1.7)	6 (8.1)	
Diverting loop ileostomy created, no. (%)			
Yes	11 (18.3)	6 (8.1)	0.129^a^
No	49 (81.7)	68 (91.9)	
Intraoperative complications, no. (%)			
Yes	3 (5.0)	0 (0)	0.876^c^
No	57 (95.0)	74 (100)	
Anastomosis, no. (%)			
ICA	60 (100)	74 (100)	–
ECA	0 (0)	0 (0)	
Method of securing anvil to the proximal bowel, no. (%)			
Pursestring suture + endoloop	60 (100)	74 (100)	–
Other technique	0 (0)	0 (0)	
Method of closing rectal cuff, no. (%)			
Pursestring suture	52 (86.7)	68 (91.9)	0.353^b^
Robotic stapler	8 (13.3)	6 (8.1)	
Mesenteric thinning maneuver, no. (%)			
Yes	44 (73.3)	15 (20.3)	< 0.001
No	16 (26.7)	59 (79.7)	
Specimen extraction, no. (%)			
Transrectal	59 (98.3)	74 (100)	0.447^c^
Transabdominal	1 (1.7)	0 (0)	
Converted to open or other MIS			
Yes	0 (0%)	0 (0%)	–
No	60 (100%)	74 (100%)	

*SD* standard deviation, *ICA* intracorporeal anastomosis, *ECA* extracorporeal anastomosis, *MIS* minimally invasive surgery

^a^Pearson’s Chi-square

^b^Independent *t*-test

^c^Fisher’s exact test

In our assessment, we identified seven technical modifications and considerations to facilitate successful completion of the NICE procedure in the background of complicated diverticulitis. The first is to detach the most inflamed portions of the disease in a lateral to medial fashion from the attachments to the lateral pelvic peritoneum and any viscus without entering the retroperitoneum. The second is to divide the mesentery close to the bowel to minimize exposure to retroperitoneal structures and preserve the superior rectal artery. The third is to use the bipolar and Vessel Sealer Extend™ (VSE) in concert to control bleeding while dividing the thickened and chalky mesentery. The fourth is to release the lateral rectal and anterior peritoneal reflection to straighten and lengthen the rectum in preparation for the natural-orifice portions of the procedure. The fifth is to prepare a NICE back table with routine use of the rectal sizers to dilate the rectum and the sixth is to assess and thin the specimen when necessary by shaving the mesentery from the surface of the bowel prior to extraction (Table 3). The final important consideration concerns closing the rectal cuff. We prefer a pursestring closure to avoid crossing staple lines; however, if the cuff is low and wide, we opt for the robotic linear stapler for closure. The steps and considerations are featured in the accompanying video.

**Table 3:** Seven key steps and considerations

Early release of the disease	Drop down of adhered portions of the disease from lateral, pelvic and visceral attachments in a lateral to medial fashion
Mesentery-sparing dissection	Divide the mesentery close to the bowel and preserve the superior rectal artery
Control the mesenteric vasculature	Use the bipolar and Vessel Sealer Extend™ in concert to control bleeding while dividing the thickened tissue and chalky mesentery
Release the rectal reflection	Release the lateral and anterior peritoneal reflection to straighten and lengthen the rectum in preparation for the natural-orifice portions of the procedure
NICE back table set up	Prepare table for transrectal extraction with small Alexis, long kocher clamp, ring forceps and medium and large rectal sizers for dilation
Thinning maneuver	Assess and thin bulky specimen by shaving the mesentery from the surface of the bowel prior to extraction
Closure of rectal cuff	Prefer closure with a pursestring suture to avoid crossing staple lines. If the rectal cuff is low and wide, closure with the linear stapler is necessary

## Discussion

During minimally invasive colorectal surgery utilization of the rectum as a natural-orifice allows one to accomplish all steps of the procedure in an entirely intracorporeal approach without an abdominal wall incision. The concept of TRSE and ICA was first described 30 years ago [1] and since that time, numerous authors have touted the benefits including less postoperative pain, lower opioid use, earlier recovery, lower surgical site infection and lower incisional hernia rates [3, 5, 12–15]. Despite these benefits, left-sided colectomy with TRSE and ICA has not gained widespread adaptation primarily due to the technical difficulties associated with this approach [15].

Enabling technologies, such as the robotic platform and modernized instrumentation, along with the strive for even less invasive approaches has led to a resurgence of interest [6]. We reported our initial experience using robotic technology and developed a stepwise approach that we named the NICE procedure [6, 8]. We have since expanded this approach to more challenging procedures such as complicated diverticulitis. These cases are technically difficult when performed laparoscopically as evidenced by conversion rates reported as high as 22.2–30% [9, 10]. The inflammatory process often results in poor visualization, distorted anatomy and peritoneal adhesions [11, 16, 17]. These factors lead to inability to identify safe planes of dissection, difficulty manipulating tissue and cumbersome bleeding [9, 10]. Furthermore, the presence of fistulas, strictures, chronic abscesses and enlarged surgical specimens have been associated with failure in extracting the specimen transrectally [18–20].

We have performed 134 resections using the NICE procedure robotic approach over a 2-year period and categorized them into complicated and uncomplicated disease. As anticipated the complicated disease cohort required significantly more operative time and these cases and were associated with higher rates of diverting loop ileostomy. Thinning of the mesentery to facilitate transrectal extraction was also required in significantly more patients with complicated disease compared to the uncomplicated group. However, we were able to successfully achieve transrectal extraction of the specimen and complete an ICA in both groups. Furthermore, there were no conversions to another MIS approach or open surgery in this series. In our prior work, we presented our early experience and focused on feasibility and a stepwise approach. In our current work, we illustrate and disseminate new concepts based on our expanded experience in patients with complicated diverticulitis. Over the course of these cases, we have modified and refined our technique to address the unique challenges in this cohort of patients and have identified seven key considerations and modifications that have not been previously described.

The first consideration is early takedown and release of inflammatory adhesions or abscess cavity along the pelvic sidewall and any associated fistula. The sigmoid colon is dropped down in a lateral to medial approach under direct visualization while avoiding the critical structures in the retroperitoneal plane. The second consideration is to perform a mesenteric-sparing approach with division of the tissue close to the bowel wall. The line of dissection is straightforward, and it yields a less bulky specimen which is beneficial for transrectal extraction. This approach also preserves the inferior and superior mesenteric artery and protect against inadvertent damage of the underlying autonomic nerves and ureter and is associated with less sexual, urinary and bladder dysfunction [21, 22]. However, this technique requires more advanced minimally invasive skills to dissect through thick, bulky and chalky mesentery which often bleeds [21, 22]. Therefore, a third critical factor is the coordinated use of the vessel sealer in concert with the fenestrated bipolar to divide the tissue and control cumbersome mesentery bleeding.

The transrectal extraction of the specimen can be one of the most difficult portions of the procedure. A fourth consideration is to routinely release the lateral and the anterior rectal reflection to help straighten and lengthen the rectum in preparation for specimen extraction. Only the thin layer of the peritoneum requires division. As with all cases of resection for diverticulitis it is prudent to ensure the distal resection is along the upper third of the rectum where the lumen diameter is larger than in the sigmoid. This has been shown to remove the high pressure zone of the sigmoid and decrease rates of recurrent disease [23, 24] but for the NICE procedure it also servers the purpose of offering a wider lumen for specimen extraction. In the event there is leakage of bowel contents from the open left colon during the extraction process, local irrigation and aspiration will be required.

A fifth consideration is the routine use of a NICE back table replete with the small Alexis wound retractor, Kocher clamp, ring forceps and rectal sizers. We begin with passage of the medium and large sizer in sequence before and after the Alexis retractor is placed. Inability to pass the large sizer corresponds with difficulty in removing the specimen. In such cases, we typically excise an additional 2–3 cm of the rectal cuff until the large sizer can readily pass.

In our series, nearly three-fourths of the specimens were too large to be extracted transrectally and a sixth important modifying technique is to thin the specimen to facilitate removal. We determine the need to thin the specimen on case-by-case basis depending on the intraoperative characteristics of the specimen size and the size of the open rectal lumen. Once we divide the rectum, we insert the medium and then large rectal sizers to help with this assessment and to straighten and dilate the rectum. We then insert the Alexis and gently extract the specimen. If we find resistance, we then opt to thin the specimen at this time. It is important to note that the presence of a malignancy should be ruled out before consideration of manipulation of the specimen intracorporeally. The thinning maneuver consists of shaving the mesentery from the bowel wall to separate the specimen in two pieces which are serially extracted. To our knowledge, we are the first to report on this technique, which has become one the most important steps to overcome the issue of bulky specimen size. We find the monopolar scissors best for dividing the mesentery from the undersurface of bowel.

A seventh consideration concerns closure of the rectal cuff. In most cases, we close with a pursestring suture which results in a circular stapled anastomosis without crossing staple lines. Avoiding crossing staple lines has been associated with lower rate of anastomotic leaks [18, 25]. However, in cases in which the rectum is divided distally and is very wide, we prefer to close the cuff with the robotic linear stapler.

There are limitations to our work. These procedures are still relatively new and the ability for widespread adaptation as well as the learning curve is yet unknown. The procedures were performed by an experienced colorectal surgeon and a dedicated robotic team. These cases require an experienced bedside assistant as well as an assisting surgeon for the natural-orifice portions of the procedure and such resources may not be available at all centers. The accompanying video is one of sixty cases in this cohort and is highly edited. Variations in anatomy from the large cohort are not represented.

## Conclusions

We illustrate the robotic NICE procedure for those presenting with complicated diverticulitis. Several modifications and key considerations are presented to overcome unique technical challenges in this cohort of patients. High rates of success can be achieved without the need for abdominal extraction incision or conversions. Larger prospective studies are necessary to corroborate our findings and validate reproducibility.

## Supplementary Information

Below is the link to the electronic supplementary material.

Supplementary file1 (MP4 305221 KB)

## References

[1] Franklin ME, Ramos R, Rosenthal D, Schuessler W (1993). Laparoscopic colonic procedures. World J Surg.

[2] Darzi A, Super P, Guillou PJ, Monson JR (1994). Laparoscopic sigmoid colectomy: total laparoscopic approach. Dis Colon Rectum.

[3] Zattoni D, Popeskou GS, Christoforidis D (2018). Left colon resection with transrectal specimen extraction: current status. Tech Coloproctol.

[4] Wolthuis AM, Bislenghi G, de Buck van Overstraeten A, D’Hoore A (2015). Transanal total mesorectal excision: towards standardization of technique. World J Gastroenterol.

[5] Leung AL, Cheung HY, Fok BK, Chung CC, Li MK, Tang CN (2013). Prospective randomized trial of hybrid NOTES colectomy versus conventional laparoscopic colectomy for left-sided colonic tumors. World J Surg.

[6] Minjares-Granillo RO, Dimas BA, LeFave JJ, Haas EM (2019). Robotic left-sided colorectal resection with natural orifice IntraCorporeal anastomosis with extraction of specimen: the NICE procedure. A pilot study of consecutive cases. Am J Surg.

[7] Scotton G, Contardo T, Zerbinati A, Tosato SM, Orsini C, Morpurgo E (2018). From laparoscopic right colectomy with extracorporeal anastomosis to robot-assisted intracorporeal anastomosis to totally robotic right colectomy for cancer: the evolution of robotic multiquadrant abdominal surgery. J Laparoendosc Adv Surg Tech A.

[8] Minjares RO, Dimas BA, Ghabra S, LeFave JJ, Haas EM (2020). Surgical resection for diverticulitis using robotic natural orifice intracorporeal anastomosis and transrectal extraction approach: the NICE procedure. J Robot Surg.

[9] Xia J, Paul Olson TJ, Rosen SA (2019). Robotic-assisted surgery for complicated and non-complicated diverticulitis: a single-surgeon case series. J Robot Surg.

[10] Maciel V, Lujan HJ, Plasencia G (2014). Diverticular disease complicated with colovesical fistula: laparoscopic versus robotic management. Int Surg.

[11] Mahmoud NN, Riddle EW (2017). Minimally invasive surgery for complicated diverticulitis. J Gastrointest Surg.

[12] Franklin ME, Liang S, Russek K (2013). Natural orifice specimen extraction in laparoscopic colorectal surgery: transanal and transvaginal approaches. Tech Coloproctol.

[13] Ma B, Huang XZ, Gao P (2015). Laparoscopic resection with natural orifice specimen extraction versus conventional laparoscopy for colorectal disease: a meta-analysis. Int J Colorectal Dis.

[14] Wolthuis AM, de Buck van Overstraeten A, D’Hoore A (2014). Laparoscopic natural orifice specimen extraction-colectomy: a systematic review. World J Gastroenterol.

[15] Wolthuis AM, Fieuws S, Van Den Bosch A, de Buck van Overstraeten A, D’Hoore A (2015). Randomized clinical trial of laparoscopic colectomy with or without natural-orifice specimen extraction. Br J Surg.

[16] Elliott PA, McLemore EC, Abbass MA, Abbas MA (2015). Robotic versus laparoscopic resection for sigmoid diverticulitis with fistula. J Robot Surg.

[17] Cassini D, Depalma N, Grieco M, Cirocchi R, Manoochehri F, Baldazzi G (2019). Robotic pelvic dissection as surgical treatment of complicated diverticulitis in elective settings: a comparative study with fully laparoscopic procedure. Surg Endosc.

[18] Saurabh B, Chang SC, Ke TW (2017). Natural orifice specimen extraction with single stapling colorectal anastomosis for laparoscopic anterior resection: feasibility, outcomes, and technical considerations. Dis Colon Rectum.

[19] Lamm SH, Zerz A, Efeoglou A, Steinemann DC (2015). Transrectal rigid-hybrid natural orifice translumenal endoscopic sigmoidectomy for diverticular disease: a prospective cohort study. J Am Coll Surg.

[20] Saad S, Hosogi H (2011). Laparoscopic left colectomy combined with natural orifice access: operative technique and initial results. Surg Endosc.

[21] Mari G, Crippa J, Costanzi A (2017). Genito-urinary function and quality of life after elective totally laparoscopic sigmoidectomy after at least one episode of complicated diverticular disease according to two different vascular approaches: the IMA low ligation or the IMA preservation. Chirurgia (Bucur).

[22] Cirocchi R, Popivanov G, Binda GA (2019). Sigmoid resection for diverticular disease—to ligate or to preserve the inferior mesenteric artery? Results of a systematic review and meta-analysis. Colorectal Dis.

[23] Francis NK, Sylla P, Abou-Khalil M (2019). EAES and SAGES 2018 consensus conference on acute diverticulitis management: evidence-based recommendations for clinical practice. Surg Endosc.

[24] Hall J, Hardiman K, Lee S (2020). The American Society of Colon and Rectal Surgeons clinical practice guidelines for the treatment of left-sided colonic diverticulitis. Dis Colon Rectum.

[25] Marecik SJ, Chaudhry V, Pearl R, Park JJ, Prasad LM (2007). Single-stapled double-pursestring anastomosis after anterior resection of the rectum. Am J Surg.

